# Carer involvement in compulsory out-patient psychiatric care in England

**DOI:** 10.1186/s12913-017-2716-z

**Published:** 2017-11-21

**Authors:** Jorun Rugkåsa, Krysia Canvin

**Affiliations:** 1 Health Services Research Unit, Akershus University Hospital, 1478 Lørenskog, Norway; 2 Centre for Care Research, University College of Southeast Norway, Porsgrunn, Norway; 3 Department of Psychiatry, University of Oxford, England, UK

**Keywords:** Community treatment orders, Coercion, Family caregivers, Carers, Qualitative interviews, Community psychiatry

## Abstract

**Background:**

There is an expectation in current heath care policy that family carers are involved in service delivery. This is also the case with compulsory outpatient mental health care, Community Treatment Orders (CTOs) that were introduced in England in 2008. No study has systematically investigated family involvement through the CTO process.

**Method:**

We conducted qualitative interviews with 24 family carers to ascertain their views and experiences of involvement in CTOs. The transcripts were subjected to thematic analysis that incorporated both deductive and inductive elements.

**Results:**

We found significant variation in both the type and extent of family carer involvement throughout the CTO process (initiation, recall to hospital, renewal, tribunal hearings, discharge). Some were satisfied with their level of involvement while others felt (at least partly) excluded or that they wanted to be more involved. Some wanted less involvement than what they had. From the interviews we identified key factors shaping carers' involvement. These included: perceptions of patient preference; concern over the relationship to the patient; carers’ knowledge of the CTO and of the potential for carer involvement; access to and relationships with health professionals; issues of patient confidentiality; opportunities for private discussions, and; health professionals limiting involvement. These factors show that health professionals have many opportunities to facilitate, or hinder, carer involvement. The various roles attributed to carers, such ‘proxy’ for patient decision, ‘gatekeeper’ to services, ‘mother’ or ‘expert carer’, however, conflict with one another and make the overall role unclear.

**Conclusions:**

There is a need for clarification of the expectations of carers in individual care situations, for carers to be equipped with the information they need to in order to be involved, and for services to find flexible and innovative ways of ensuring continuous, open communication. The introduction of CTOs in England has not been successful in its ambition for carer involvement.

**Electronic supplementary material:**

The online version of this article (10.1186/s12913-017-2716-z) contains supplementary material, which is available to authorized users.

## Background

### From family to carer: The role of family members in modern health care

Deinstitutionalisation of health services has shifted significant care responsibilities onto patients’ families. As a policy it relies to some extent on cultural obligations for family members (particularly women) to look after one another [1, 2]. There is now increased focus on families as partners in service delivery and their home as a locus of care. Health professionals are often encouraged or even required to involve patients’ family members in decisions [3–5]. There has been a gradual development towards labelling the assistance family members provide for one another as ‘care’ and those performing it ‘carers’. The carer role was first recognised in law in *The Carers (Recognition and Services) Act 1995*. Subsequent legislation and policy has clarified complementary rights and responsibilities for carers, services and authorities in the delivery of health care. A national Carers’ Strategy 2008–2018 [6, 7] commits professionals to view carers as integral parts of health care systems, involve them as a partner in service delivery, and recognise their ‘carer expertise’:

While the person being looked after is usually the expert in their own care, the carer too is a real expert. That being the case, carers should be consulted as partners in care and their unique knowledge and expertise recognised. [6, p 38]

### Family carers’ role in coercion in outpatient mental health services

A role for family carers in the planning and execution of formal compulsion has long been established and is usually written into mental health legislation. This is often in the capacity of ‘Next of Kin’^1^ which entails certain rights to initiate or end involuntary treatment. Three partly overlapping roles for carers have been identified in mental health legislation. First, they may act as ‘gatekeepers’ who monitor patients and decide when professional intervention (including compulsion) is required. Second, they may serve as a ‘proxy’ for patients who lack decision making capacity. Third, carers are portrayed as ‘advocates’ working in the interests of service users by representing their wishes [8]. The implementation of mental health laws is thus to some extent premised on the participation of family in the coercion of patients. This can sometimes place family members in adversarial positions vis-à-vis each other [9, 10].

Carer involvement is also specified for the implementation of Community Treatment Orders (CTOs), which were introduced into the Mental Health Act for England & Wales in 2008. CTOs are intended to ensure that patients with severe mental illness get the care they need, including early intervention during relapses, by making adherence to treatment plans a legal requirement when patients are treated in the community. Similar legal regimes exist in around 75 jurisdictions across the world as a response to deinstitutionalisation of mental health services, which created a need for compulsion outside of institutions.

**Fig. 1:**
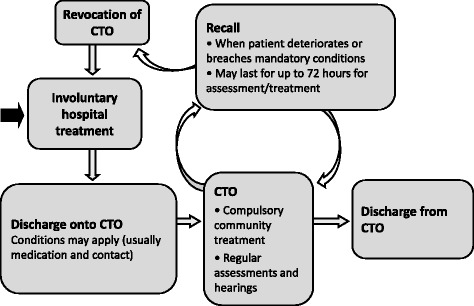
The CTO process in England and Wales

The CTO regime in England and Wales allows for swift *recall* to hospital when there are signs of deterioration. After a recall (which may last up to 72 h) the patient returns home on the CTO or the order is *revoked* and he or she remains in hospital under compulsion (See Fig. 1 and Table 1 for details). Specific CTO conditions may be written into the order, and nearly all oblige patients to take medication and staying in contact with services. Some stipulate that patients must reside at a particular address, and a range of other requirements are also occasionally specified [11]. Family carers’ role in the England & Wales regime is described in the *Code of Practice* accompanying the legislation:

*Particular attention should be paid to carers and relatives when they raise a concern that the patient is not complying with the conditions or that the patient’s mental health appears to be deteriorating. The team responsible for the patient needs to give due weight to those concerns and any requests made by the carers or relatives in deciding what action to take. Carers and relatives are typically in much more frequent contact with the patient than professionals, even under well-run care plans. Their concerns may prompt a review of how [the CTO] is working for that patient and whether the criteria for recall to hospital might be met. The managers of responsible hospitals should ensure that local protocols are in place to cover how concerns raised should be addressed and taken forward* ([12] paragraph 25.46).

As is clear from this excerpt, carer involvement is central to how CTOs are intended to work.

**Table 1:** The CTO regime in England and Wales

Criteria	CTOs in England and Wales are initiated by a psychiatrist and a social worker. The legal criteria includes that the patient suffers from a mental disorder for which they require treatment to protect their health and safety or that of others, that the treatment (which must be available) can continue in the community, and that the patient is liable to be recalled to hospital. A CTO can only be made when the patient is already detained for hospital treatment.
Conditions	All CTOs have two mandatory conditions. These require the patient to make themselves available for assessments (i) when an independent medic is assessing the appropriateness of treatment and (ii) when renewal of the CTO is considered. In addition, discretionary conditions may be specified in the CTO form based on the knowledge of an individual patient. The most common are requirements to take medication and stay in contact with services. Having to live on a specified address, comply with monitoring of blood levels and abstain from drugs and alcohol are also used in many CTOs.
Recall and revocation	Should the patient breach a mandatory condition or deteriorate, he or she may be *recalled* to hospital for up to 72 h, after which the order can be *revoked* and the patient remains in hospital for involuntary treatment, they continue on a CTO in the community, or they are discharged from compulsion altogether.
Renewal	The CTO lasts initially for six months. They can be renewed for a further six months and then for 12-month periods.
Discharge	The orders can be ended at any time by the responsible psychiatrist. They can also be ended in a judicial hearing. The patient has the right to appeal to managers of the treating hospital and to one hearing by the Mental Health Review Tribunal (MHRN) in each CTO period. Routine hearings are held to ensure that the legal criteria for the CTO are still met even if the patient does not appeal.

A search of the international literature (using *Ovid, Web of Science, Medline, Sociological Abstract, Embase* and *CINAHL*) identified a small number of studies of carer experiences of CTOs. Most indicated that carers in general welcome CTOs insofar as they facilitate support to the patient. Carers, along with patients and psychiatrists, see the key justification for CTOs as obliging patients to take medication and to stay in contact with services [13, 14]. Some studies include information on carer involvement in CTO. There is a suggestion that although some carers become more involved under the CTO, many experience insufficient consultation, unmet information needs, and are not listened to or ignored [4, 15–17]. Some studies indicate that that carer involvement may harm family relationships [4, 5]. A lack of understanding of the legal mechanisms on behalf of carers has been observed [13, 18]. No study has systematically examined carer involvement at different stages of the CTO process or the factors shaping their involvement. To fill this gap in the literature, we report on qualitative interviews with 24 carers across England. First, we outline their experiences of involvement throughout the process and, second, we identify factors that can explain observed differences in levels of involvement. In the Discussion we then assess how these experiences measure up to the ambitions set out in public policy.

## Methods

We report data from a large qualitative study of CTO experience of patients, carers and psychiatrists that forms part of the *Oxford Community Treatment Order Evaluation Trial* (OCTET) research programme on coercion in community services [19].

### Sample

In-depth qualitative interviews with 24 family carers of patients with experience of CTOs were conducted in 2012. There were three couples among the participants so that the sample collectively had experience of 21 patients on CTO (18 of whom were male). To achieve a purposive sample [20] with variation in the characteristics and experiences indicated in Table 1, we contacted around 40 local and national carer support organisations across England that spread information about the study widely to carers they were in contact with. Twenty of the participants contacted us as a result of getting information through an email or newsletter, and four were recruited when we gave presentations at carer events.

### Data collection

All interviews were conducted by the first author. The topic guide (see Additional file 1) comprised questions about experiences and views of CTOs. Everyone was prompted about their involvement in the various stages of the CTO process, including changes over time and circumstances. Interviews lasted on average 90 min (range 55–135 min). All interviews were digitally recorded, transcribed *ad verbatim* and the transcripts were checked against the recording for accuracy.

### Analysis

The study employed a modified version of Grounded Theory [21], a qualitative methodology that analyses data in cycles of induction and deduction. Exploratory qualitative studies usually address research questions in staged analyses. Given the aim of the overall programme, the first analysis was a broad, open exploration of carers’ experiences of CTOs mechanisms and their impression of how they worked, which was juxtaposed with patients’ and psychiatrists’ experiences [13].

The present article reports the second strand of analysis of the data from the interviews with carers. As we specifically wanted to investigate their involvement in CTOs, the initial step in this second analysis took a deductive approach by coding for (JR) and analysing (JR and KC) predetermined topics, including participants’ involvement in CTO initiation; monitoring of patients on CTO; recalls, and; renewal. These data are presented in the first section of Results. The second analytical stage took an inductive approach. We re-read all data to identify topics that could explain *how* carers were involved and *why* their involvement varied. These topics were then used in a second round of focused coding (JR). The subsequent thematic analysis (JR and KC) [22] led to the development of the themes reported in the second section. In the presentation below, quotes from the transcripts are included to illustrate and validate our interpretations [23].

### Ethical issues

Ethical approval was given by Staffordshire NHS Research Ethics Committee (ref. 08/H1204/131). All participants provided written consent to take part and for the interview to be audio-recorded. They were given the opportunity to review and edit their transcript before giving separate written consent for the use of direct quotations. All names of people and organisations have been deleted or altered, except the interviewer’s.

## Results

The sample of 24 consisted mainly of parents of patients on CTOs. One was the sister and one was the husband of someone on a CTO. The sample lived across England, and collectively they had experience of all the different parts of the CTO process (see Table 2). None of the participants had objected to their relative being placed on a CTO. In general, they welcomed CTOs as a new approach to ensure patients achieved more stability by accepting the help they needed and staying out of hospital.

**Table 2:** Characteristics of the sample and CTO experience at the time of interview

			Carers ^a^ *N* = 24 (Patients *N* = 21)
Gender	Male		7
	Female		17
Ethnicity	White		21
	Black		0
	Others		3
Geographical location	North West		3
	South West		4
	South East		8
	East		1
	East Midlands		1
	West Midlands		2
	London		3
Relationship to person cared for	Parent		22
	Spouse		1
	Sibling		1
Patient’s diagnosis and condition	Schizophrenia/schizoaffective disorder		(20) ^b^
	Bipolar		(1)
	Depot medication		(11)
	History of violence		(12)
Patient’s CTO status at interview	Ongoing CTO		(14)
	Revoked		(2)
	Discharged		(3)
	Unknown		(2)
	Experience of recall		(8)
Duration of CTO	<6 months		(1)
	6–12 months (renewed once)		(9)
	12+ months (renewed twice or more)		(8)
	Unknown		(3)

^a^Including 3 couples

^b^Two carers disputed the diagnosis of schizophrenia

### Experiences of carer involvement through the CTO process

#### Carer involvement in the making of CTOs

Some level of carer involvement was seen by the participants as necessary when a CTO was imposed because their close involvement in the life of the patient meant they possessed detailed knowledge about the situation. This was particularly the case when carers and patients lived together. Few reported that there had been any real discussion about the CTOs and many had limited information about the order when it was imposed. Although some had taken part in all or most of the relevant meetings and been involved in discussions and decisions, generally participants had been informed but not actively involved in decision making. Many used the term “consulted” and were happy with that level of involvement. Some said not having to be part of the decision of making the CTO had been a relief:

Carol: They said to my husband “if you [object] we’ll go to court and we’ll get the order”, so it was out of our hands

**Jorun: Is that a good thing that it’s out of your hands?**

Carol: Yes, definitely. Definitely. Definitely. I wouldn’t wish this on anybody*.*

Others were disappointed that communication was limited. Jenny, who had been in favour of the CTO, was frustrated that the process of placing her son on it had taken nine months, and neither she nor her son had received much information during that time.

#### Carer involvement in monitoring patients on CTO

Many perceived that the effort they put into monitoring patients’ medication adherence and communicating with the mental health team was important for the CTO to “work”.

Lakshman: [The team was] supposed to come at 10 o’clock. They didn’t come. 10.30 - nothing happened. So I rang the people: they’re down the road. I said “nobody has come. I’m going to give the medication because he’s got to go to sleep”

Some reported very good collaboration with services. Clare and Steve routinely called their son’s team to discuss concerns, and the team was usually quick to react. Others were grateful that responsibilities for monitoring patients had shifted somewhat from carers towards health professionals under the CTO:

Jeff: And it puts the onus on the team as well because obviously he’s monitored and it takes it off us and puts it onto them which is good. Because the way it was before it was us, and when we highlighted [that he was] ill and action hasn’t been taken quick enough and it’s ended up where he’s become really high*.*

Some participants had negotiated with health professionals over medication, either together with or on behalf of the patient. Lindsey and Jeff, for instance, had reached an agreement to replace their son’s depot medication with oral medication monitored by their local chemist. Others had attempted such negotiation, but not succeeded because “the health professionals did not listen”.

Many were concerned that there was insufficient professional supervision of patients on CTO. Jenny's son had only been seen once by his psychiatrist and twice by his care-coordinator in six months, which she said was insufficient. Others complained that home visits were too brief and superficial to detect the patient’s condition:

Sarah: They came to the door and they said “Hello [patient name] are you all right?” And he said “yes”. “Okay. Bye, bye” and they went. That was it and what good is that? They may as well not go*.*

As a result, Sarah and the health professionals sometimes came to very different conclusions as to her son’s situation:

Sarah: I went around to his house and as soon as he opened the door I knew he was drunk and I went in and there was an empty whisky bottle on the table, the kitchen was full of smoke […]We were going out for a meal and he is quite menacing when he is drunk and so I said “you are drunk and I am not taking you”. He got really quite nasty with me and he ran after me and grabbed hold of me and I managed to get in the car and I had to quickly lock it and he was actually hanging on to the car as I was driving up the street and so I was really upset. I phoned the team and said “look, I have just been around to [patient] and he is really drunk and he has been very aggressive”. I spoke to one of the nurses who said “well we have just been around 10 minutes ago and he was fine”. I said “he is not fine, he is drunk! His breath reeks!” “Well we don’t get close enough to his breath”*.*

It was explained that due to such insufficient supervision by services, or a lack of assistance beyond ensuring medication was taken, family carers sometimes needed to take on more responsibilities than they thought was fair:

Niamh: I’d want to have less [involvement] but I’ve got too much now because every time they [make changes] more comes back on me. And because of all this, it’s all come onto me to go instead of [the patient], to talk to [the care worker] about it. Where’s the worker to do that? […] He hasn’t got anybody to chat it over with or have coffee so I have to have him Sunday, I have to go out in the week, I have to take him shopping, I have to do his washing, I have to do his ironing. And I’m disabled. I’m disabled. I said “I don’t want all this. I don’t want all this. He might tell you that I enjoy doing it but I don’t”*.*

While services often expected carers to monitor medication, they did not always collaborate with carers’ strategies to enable adherence. Jenny, whose son really disliked being at the depot clinic, had tried to call up in advance of her son’s appointment so they could take the medicine out of the fridge and thereby shorten the time he needed to be there, but, she said, they would not do it. Sarah’s son simply needed assistance with blister packs and could miss tablets unless she marked on the pack where he should begin. She said staff could easily do this instead of her if they took the time.

#### Carer involvement in recall to hospital

Overall, the *recall* mechanism was perceived to be the main advantage of CTOs because it meant services could intervene early instead of having to wait until the patient had completely deteriorated and family carers were stressed out. Compared with pre-CTO MHA assessments, the power of recall required less family involvement in legal procedures during relapse, and this reduced stress significantly. Nevertheless, many were, and wanted to be, involved in decision making surrounding recalls, and several reported positive experiences of this. For example, Rose had contacted services when her son showed signs of deterioration and together they managed to encourage him to accept a short voluntary hospital stay, avoiding the more intrusive formal approach. Naomi’s request to halt a recall because the only available bed was some distance away was accepted by the team and they agreed that instead she would monitor her son more closely than usual. Liz said three of the four recalls initiated when her daughter had not turned up for depot appointments had in her view been premature, and she had been instrumental in getting them stopped.

Others felt ignored during relapse. Despite positive experiences the first two times, Rose said the poor communication between health professionals and both her son and she had contributed to the failure of his third recall:

Rose: [They] said to [patient’s name] on the Thursday “we’ll come back tomorrow morning with your depot injection”. When they went back on the Friday they got no answer. That’s when the recall was initiated and we later discovered that on the Friday he’d already been to the bank, drawn out every last penny he had and taken himself to London to get a ticket to go to Spain. And I swear that that was, you know, it was not the right way to handle him but as I say at that point in time we didn’t seem to be listened to by his consultant anyway*.*

#### Carer involvement in CTO renewal and discharge

There was also variation in carers’ experiences of involvement during CTO renewal. Many supported the renewal and thought the CTO needed to be in place for a considerable time. Most were simply asked for their opinion during this process, but others had more substantive involvement such as attending and speaking at renewal meetings or tribunals.

Some were unhappy that the health professionals did not pay attention to their views. Sarah said she had been ignored when she disagreed that the CTO should be renewed because she did not think it was working:

Sarah: So what [the psychiatrist] said at the last meeting was “I am going to keep you on your CTO.” And I asked why because I said “to be honest with you I don’t want him to go back into hospital [through recalls]. What happened last time was awful and I would really object to that and so I don’t see the point.” [The psychiatrist said:] “No, no it’s better”. And she wasn’t taking him off it. We did phone [national mental health charity] and they said I can write and object but I don’t want to offend [the psychiatrist]*.*

Some complained that renewals were made on the basis of inaccurate or erroneous patient records. Jenny said her son’s CTO renewal had been made on the basis of out-dated information. She supported him in appealing and eventually getting the order removed.

### Factors shaping variation in carer involvement

As just shown, there was variation in how family carers were involved in the CTO process. In this section we explore some of the factors shaping this variation, based on themes from the interviews.

#### Perceptions of patient preference

Most participants said that their relative wanted them to be actively involved by taking part in interactions with services and by giving practical and emotional support. Others said the patient did not want them involved, especially during psychotic episodes: Liz’s daughter never objected to her involvement when she was doing OK but once, when severely unwell, she had insisted on a DNA test before accepting her mother’s involvement. Tanya said her son was more accepting of her communicating with services now that he was on a CTO, and to preserve this level of involvement she always informed in him before she did so:

Tanya: Before he was sectioned and he was very, very paranoid, he didn’t want me anywhere near anything to do with mental health. But since he’s been on the CTO he’s been more amenable to me being involved. I tell him each time I’m going to do anything so he does know what’s happening. And at the moment - touch wood - he’s ok. As long as I tell him what it is that I’m going to talk to the psychiatrist about or CPN about*.*

#### Concern over the relationship to the patient

Carer involvement in the CTO process could cause conflict in their relationship with the patient, and especially so if the carer had initiated the CTO or supported recalls or renewals. In Ray’s case there was a long history of his wife thinking he was “in cahoots” with services and her objection to his involvement was not reduced under the CTO. Even if he thought she was now more stable he was still concerned she might turn violent when he, for example, spoke up at Tribunals. He therefore saw his involvement as a threat to their relationships. Such concerns meant carers sometimes needed to make tough decisions about prioritising between short term impact to the relationship and longer term impact on patients’ health:

**Jorun: So he wanted to be off it and you were there ‘in court’ saying you wanted him to stay on it?**

Tanya: Yes. I did talk to him before about it and yes, he found it very difficult.

**Jorun: How did you find it?**

Tanya: Difficult. Because you want to do right by what he wants but you have to look at what I thought was best for him in the long term and I still think it was the right thing to do, definitely*.*

Naomi described a similar conflict trying to maintain a good relationship with her son while also being his Next of Kin. To resolve this, the family changed their strategy by letting her other son take over the legal role while she remained his day-to-day carer.

Carers and patients commonly disagree about the benefit of adhering to prescribed medication [24]. Some participants found it very helpful that the CTO placed an obligation on the patient to adhere to medication from “the outside”, with the authority of the law. By taking this responsibility away from them, the CTO was perceived by some as protecting the relationship and thus enhancing their opportunity to get involved in other aspects of the patients’ lives. As Liz said: “it’s nice just being mum!”

#### Carers’ knowledge of the CTO and the potential for carer involvement

The delivery and receipt of information was another issue affecting how carers were involved, and again there was variation in experiences. While some reported being well informed by services, voluntary organisations or through the internet, others said they had received limited, “virtually nil” or no information. Not receiving the relevant legal paperwork, not knowing how long the CTO was going to last, and not knowing the procedure for ending it were mentioned. Insufficient information made it difficult to know what to expect and how they could contribute themselves:

Jenny: Well, I mean, I think it would have been very helpful if there had been a meeting in February when it was imposed fully to have the opportunity to talk it through to find out how long it was going to be for, what it really meant in terms of [patient’s] responsibility to himself, to the consultant, to the nursing staff. What I should, could, possibly would do in support of all of that*.*

While some displayed good understanding of the CTO process, others were unaware of, for example, the consequences of breaching conditions and its connection to recall:

Jenny: No, well, I mean, it wasn’t clearly spelled out to him. I couldn’t get a clear grip on that “if you don’t do A then B will happen”, what B was. That they would within 24 hours come and chase him? Or that they would call him on the phone and say you missed your injection and would you please come up for it and if he didn’t they would then send him [to hospital]? All of that was very unclear*.*

Consequently, Jenny was unable to explain the working of the CTO to her son who was confused about what was expected of him. Not knowing how the CTO mechanisms works also impacted on carers’ communication with health professionals about the patient’s situation. There were some examples of carers having been given inaccurate information about the CTO procedure, such as what conditions could in principle apply or how conditions or medication could be changed.

#### Access to and relationships with health professionals

While a few participants had good access to the responsible psychiatrist, most said that it was hard to get to speak to them. Clare believed this had been made even harder with the CTO. While she saw the reduced need for her being involved under the CTO as a good thing, it resulted in fewer opportunities for relationship building with the psychiatrist, making it harder to be involved when she was needed:

Clare: I’d like to meet the psychiatrist and we haven’t had that opportunity because the Community Treatment Order protects the carers but they don’t have any input in between assessments*.*

Although not always seen as a satisfactory strategy, many used the CPN as a go-between or messenger in order to communicate with the psychiatrist:

Ray: Well I will send my messages through the community psychiatric nurse who comes and sees [my wife] every two weeks and he will feed back. I mean it’s very difficult speaking directly to the consultant because the only time I can see him really is when she is in the room*.*

Carer involvement under a CTO was seen to depend on the attitude of, or relationships with, individual health professionals:

Lisa: Also I do find it’s very much up to the individual professional, whether it’s the psychiatrist, the social worker or the community nurse. Some of them seem to like you and talk to you and you feel you are working in a partnership. Others don’t. I mean I have had occasions when I’ve been glared at. That glare meant “get out of the room so I can talk to [patient]”, but instead of saying “would you leave us in privacy?” - I mean, I’m in my own house!

*[…]*

**Jorun: And you also mentioned sometimes it feels like it’s more of a partnership. What kind of examples do you have of that?**

Lisa: There used to be a great social worker. He used to ring me up and say did I think [patient name] might be relapsing and I could ring him up and say the same and we’d compare notes as to [patient name]‘s behaviour in the last week or so and say “yeah he’s going again”, something like that. Others just wouldn’t dream of ringing you up at all and once I rang up and was told “oh, we’re always aware of [patient]‘s condition, thank you”. It just depends on the individual*.*

The high number of staff involved, rapid turn-over and constant service reorganisation also impacted: building good relationships takes time, and constantly having to “go back to square one” was experienced as frustrating and as preventing on-going communication.

#### Issues of patient confidentiality

Issues surrounding patient confidentiality were frequently perceived to influence how open health professionals were to carer involvement. Some participants experienced that services simply assumed the patient would not want them involved. Sarah got her son to write a letter giving her permission to speak to his consultant. She felt she needed his explicit permission to be able to be properly involved in lobbying for her son’s medication to be reduced. Rose’s son did not want her involved with services. She believed, however, that given her role in helping him in his everyday life she had a right to be given some information about his treatment and said there was a duty on health professionals to find ways to get this information to her:

Rose: I do understand that maybe it’s a case of it’s been suggested to [patient’s name] “do you want your mum to come along to the appointment” and he’s probably said “no”[…]We’ve had problems over the years and I do understand, yes I do understand the very basic need for confidentiality but I think it’s taken to far too much of an extreme. I think there are certain things that don’t necessarily need to be kept confidential.

**Jorun: Such as?**

Rose: Such as if you’re dealing with carers that have a large input into the treatment they have every right to know what medication is being taken, when it’s being taken, what sort of other treatment is involved*.*

#### Opportunities for private discussions

As Ray’s statement above suggests, he found it difficult to fully express his view if unable to speak to health professionals without his wife being present. If the carer held a very different view to the patient this could risk not only damage to their relationship but also endanger future involvement:

Sarah: I am going to try and talk to [the psychiatrist] without [patient name] there where I can talk more honestly. Because when he is there I can’t tell her the negative things because then he feels really criticised […]. He doesn’t want to [see the psychiatrist] more often than six months and so if I said “no I think you need to see him sooner and monitor his medication”, [patient name] would have been really angry with me and wouldn’t let me go with him to the next appointment and so now I am in a kind of a trap*.*

Participants believed services could be more imaginative in addressing such dilemmas. Some emphasised they had been “lucky” to find creative ways to deal with this and that the structure of the CTO helped:

Naomi: I think [the CTO] does make it easier because I can go and see the psychologist or his care coordinator, and I can say “I don’t want you to tell [patient’s name] but I do think things are slipping”. And that gives me a breather from ringing and saying we need the Mental Health Act. And it means three people don’t pile in, upsetting [patient]. It means that the psychologist may phone him or drop a note through the door. Yes it does make it easier. But it’s also made easier by the psychologist agreeing not to say “hello [patient], your mother rang me, I hear you’re not too well”. And I’m very lucky there*.*

#### Health professionals limiting involvement

There were a small number of examples of how health professionals attempted to limit carers’ involvement in patients’ everyday life. Two carers reported that health professionals encouraged patients to move out of the family home to live independently under the CTO, and this was experienced as pressure for reduced carer involvement. Ann said her son at one point had been threatened with a CTO if he did not move into a charity hostel. There were also one or two reports that health professionals had included statements in CTO reports that there had been carer involvement where there had been none; this was experienced as very unfair.

## Discussion

### Carer involvement in the CTO process varies

There is considerable variation in whether, to what extent and how carers are involved in the different parts of the CTO process. Some carers monitor adherence to medication and conditions or are in close collaboration with the treating team about the need for recall or admission. Many perceive their involvement as potentially contributing to how CTOs work, particularly through providing everyone involved with the right information at the right time. Some said the CTO has led to increased involvement and influence over decisions, and this is also reported in other studies [4, 14, 16, 18]. However, many carers also experience being excluded, inadequately consulted, having their views ignored, or receiving insufficient information, including legal documents. Again, this is reflected in international studies [4, 5, 14, 16, 25].

There were different views among participants regarding what constitutes the *right* level of carer involvement. Some wanted a more central role in the CTO process, while others believed this was the responsibility of health professionals. There were positive and negative experiences with reduced, increased or unchanged carer involvement under the CTO. Some of those who said they were actively involved wanted even closer involvement while others experienced increased carer involvement as a consequence of the CTO as unfair. Some were happy that services took some of responsibilities off them [4, 5, 15] and others felt excluded.

It is important to keep in mind that some carers want to be *less* involved in the care of their relative, especially in relation to medication, to allow them to focus on more positive aspects of their relationship. Yet some feel obliged to take on more responsibilities than they think is right because they do not trust the quality of the services provided. It is also noteworthy that carer involvement in CTOs is not limited to that which is conducted in *partnership* with health professionals. There were a number of instances where carers involved themselves to seek to change decisions about medication, recall or discharge, going against medical advice.

The wide variation carer involvement seems to be shaped by a range of factors, some of which are rooted in the private sphere, such as perceptions of patients’ preferences or concern over relationships with patients. Most of the factors reported here, however, are largely within the remit of health services. These include carers’ access to and communication with health professionals, information about the CTO legal framework, issues surrounding patient confidentiality, opportunities for private discussions, and ways in which health professionals might directly limit carer involvement. These factors are interlinked: issues of confidentiality feed into opportunities for private discussion, which in turn may be linked with both access to information on the one hand and potential for damaging relationships on the other.

### Carer involvement and role conflicts

Carers hold, or are expected to hold, a range of different roles. These include the roles of *gatekeeper, proxy and advocate* attributed to carers in mental health law, as mentioned in the Introduction [8], and that of ‘expert carer’, which underpins current policy. In addition, roles based in kinship and love such as *mother, father, spouse or significant others* will often, from carers’ point of view, be those experienced as most valuable and important [24].

The factors identified above contribute to potential conflicts between these variousroles. The role of a *gatekeeper* initiating a recall may conflict with role of an *advocate*, speaking on behalf of a patient who does not wish to engage in treatment. While serving as *gatekeeper* could align well with the role of *expert carer*, both these roles may conflict with that of *mother* (with expectations of unconditional support), have adverse effects on family relationships and limit future involvement. The role of *advocate*, often as a *Next of Kin* could increase carer involvement, but might in practice only be possible if the carer supports the patient’s views and judgements [16] thus precluding carers from contributing with their *expert carer* knowledge. Refusing the role of *proxy* may equally harm relationships [4]. Being an effective *advocate* for patients requires considerable knowledge and voice, which many carers say they lack. Also, as Tanya suggested above, it can be impossible simultaneously being your son’s *advocate*, who speaks up for his short term wishes and an *expert carer* who thinks longer term outcomes should be prioritised. Lack of information, issues of confidentiality and access to health professionals can limit both the *advocate* and *expert carer* role. Moreover, both these roles may convince carers to go against the view and opinion of the clinicians, thus endangering the role of *partner* in service delivery. In brief, a lack of clarity and agreement of what should be carers’ priority when being involved in service delivery, together with myriad potential or real conflicts between the various roles, can complicate and prevent their involvement.

### Unfulfilled policy ambitions for carer involvement

The Mental Health Act Code of Practice sets out a specific policy ambition for carer involvement in CTOs, as shown above. Clinicians are instructed to pay attention to carers’ requests; carers’ concerns should prompt reviews, and; there should be local protocols for how to take their involvement forward [12]. The contribution of carer expertise is also recognised. In the experience of many (though not all) carers interviewed in this study, these ambitions have not been fulfilled. In their view, services need to be better at providing carers with information throughout the CTO process, and to improve the ways in which they communicate and build relationships: carers should not have to rely on “luck” to get to speak to clinicians about treatment activities in which they are expected to be involved, including some that might take place in their own home. From carers’ perspectives, more flexible approaches are called for that, for example, can uphold patient confidentiality while also preserving good carer involvement. This issue has been recognised and a review of English policies for involving mental health carers when patients withhold consent identified 56 relevant policies and 35 supporting documents. Of those, 20% addressed information-sharing specifically and only 5% gave practical advice [26]. Local practice seems thus seems often to lag behind current policy drives and some way off that expected in public policy and law [17, 27].

### Limitations

Our sample had a good spread of age, location, gender and experiences of health services and CTO, minimising the likelihood that findings are associated with particular backgrounds, settings or NHS Trusts. It consisted of carers who volunteered to take part, and who were in contact with a local carer service. The majority were parents of CTO patients, and only three people with an ethnic minority background took part. It is possible that findings would have differed if the sample had included, for example, more siblings or those not in contact with carer services.

When we report carers’ accounts of patients’ or health professionals’ views or experiences these should not be inferred to be accurate renditions. Nevertheless, carers’ interpretations of these accounts are important because they help us to understand their own perspectives.

## Conclusion

Carers’ experiences of being involved in the CTO process vary. We have identified some factors shaping this variation, some of which surround family relationships, yet all of them are relevant to the practice of mental health professionals, who are under obligation to maximise carer involvement.

It may be unreasonable to rely on carers holding roles that are internally conflicting; it certainly seems to complicate how carers may involve themselves and sometimes have negative impact on family relationships. From our findings there seems to be an urgent need to clarify the expectations of carers in individual care situations, for carers to be equipped with the information they need to in order to be involved and for services to find flexible and innovative ways of ensuring continuous open communication. Overall, our data indicate that the introduction of CTOs in England has not been successful in its ambitions for carer involvement. Questions regarding what it is reasonable for society to expect from family carers in a time of deinstitutionalisation and diminishing budgets remain unanswered.

## Additional file

Additional file 1: Interview topic guide: OCTET carer interviews (DOC 33 kb)

## Abbreviations

CTO: Community Treatment Order
MHA: Mental Health Act for England & Wales
OCTET: Oxford Community Treatment Order Evaluation Trial

## Footnotes

1 Various terms are used in legal frameworks (e.g., Nearest Relative, Next of Kin, Named Person) to identify a person with specified rights to make decisions in relation to patient care, including in some instances proxy decisions when the patient lacks capacity.
