# Supplementary material for: Carer involvement in compulsory out-patient psychiatric care in England
Source: BMC Health Serv Res. 2017 Nov 21;17:762. doi: 10.1186/s12913-017-2716-z (PMC5698997; doi:10.1186/s12913-017-2716-z)
Supplement: Additional file 1: — Interview topic guide: OCTET carer interviews (DOC 33 kb) [file 12913_2017_2716_MOESM1_ESM.doc]

**Interview topic guide: OCTET carer interviews**

In the first part of the interview the interviewee will be invited to tell their story freely on the basis of a very open opening question:

“As you know I’m interested in hearing about your experiences of being a carer of someone on a CTO. To help me understand better it would be helpful if you could first tell me the story about how the situation came about and how it has been. Take as long as you like and after you’ve finished I’d like to return to some of the issues you raise and also some questions that I ask in all the interviews.”

The second part will return to key issues in this narrative, plus the following topics:

1. **Background to the care situation**

If not covered in the opening narrative check for:

- Family history (constitution, migration, main events)
- How the caring situation emerged
- Why the carer (as opposed to others) is in the caring role (choice, obligation, expectation)
- Perceived reasons behind the mental health problem
- Impact of the mental health problems on the family

Check facts:

- Does the patient live in the household
- Household constitution
- Other care duties
- Employment situation

1. **Involvement in CTO process**

- Why the CTO was needed
- How the CTO come about
- Carer’s involvement when the CTO was made
- Carer’s involvement in possible recalls
- Carer’s involvement in possible readmissions
- Carer’s involvement in possible renewals or discharge
- Experience of change in the carer’s involvement with treatment since the CTO
- Description of interactions and relationship with the mental health professionals involved
- The conditions of the CTO, if known
- Agreement with CTO conditions

1. **Impact of CTO on the carer role**

- What the carer role entails
- Impact on life of carer role
- Experience of being a carer in the context of the MHA (sectioning etc)
- Changes to carer role since/during the CTO (if at all)
- Things experienced as easier and/or harder with the CTO
- Counterfactual: how would care role be if CTOs were not available

1. **Patient outcomes**

- Perception of patient improvement (or not) on CTO
- If better, what about the CTO that has led to improvement
- Counterfactual: how would patient be if CTOs were not available

1. **Support for the carer**

- Support available to carer
- Support from the NHS
- Offer of State support
  - Cares’ Assessment
  - Carers’ Allowance

1. **Leverage/pressure**

- Perception of what the care-recipient needs to do to get better (prompt for medication)
- What the carer does to make him/her do those things
- What kind of conversations/interactions the carer has with patient regarding
  - Money management
  - Children/childcare
  - Housing
- Experience of whether the CTO changed the carers ability to influence patient to do things that s/he would benefit from but is reluctant to do

1. **Opinions**

- Carer’s description of what a CTO is to someone who doesn’t know what it is
- Overall evaluation of CTOs
- Perceptions of how carers’ contribution of care in the community fit into the system (interactions health care, the State and families)
